# Editorial: Host-pathogen interactions at the blood-brain barriers

**DOI:** 10.3389/fcimb.2024.1412140

**Published:** 2024-04-23

**Authors:** Christian Schwerk, Caroline Coisne, Stefan Mogk, Xiangru Wang

**Affiliations:** ^1^ Pediatric Infectious Diseases, Department of Pediatrics, Medical Faculty Mannheim, Heidelberg University, Mannheim, Germany; ^2^ Eurasanté, Lille Métropole, France; ^3^ Interfaculty Institute of Biochemistry, University of Tübingen, Tübingen, Germany; ^4^ National Key Laboratory of Agricultural Microbiology, College of Veterinary Medicine, Huazhong Agricultural University, Wuhan, China

**Keywords:** blood-brain barrier, blood-cerebrospinal fluid barrier, host-pathogen interaction, central nervous system disease, immune response

The human brain requires a well-defined homeostasis for proper operation. To guarantee undisturbed function, specific barriers separate the central nervous system (CNS) from the rest of the human body. These so-called blood-brain barriers include the “classical” blood-brain barrier (BBB), which is formed by the endothelium of the brain microvasculature, supported by astrocytes and pericytes, and the blood-cerebrospinal fluid barriers (BCSFBs), which are located at the epithelium of the choroid plexus (CP), the outer layer of the arachnoid membrane, and the blood vessels in the subarachnoid space. Taken together, these form a “physical barrier” by preventing a paracellular passage between cells, a “biochemical barrier” by specific expression of cellular transporters, and an “immunological barrier” by regulating the traffic of immune cells into the CNS.

Although the BBB and the BCSFB are highly successful in their task to serve and protect the human brain, various pathogens have developed strategies to overcome these barriers to gain entry into the CNS. Once there, these pathogens find an immune-specialized space that offers them an advantage for survival and propagation, ultimately resulting in host diseases such as meningitis, encephalitis and meningoencephalitis. To enter the CNS, the pathogens will undergo specific interactions with host cells at the BBB and the BCSFB.

Coxsackie viruses are considered one of the leading causes of aseptic meningitis. Mamana et al. infected stem cell-derived brain-like endothelial cells (iBECs) with the clinically relevant Coxsackie virus species B3 (CVB3). Interestingly, although transendothelial electrical resistance (TEER) decreased later during infection, iBEC monolayers remained morphologically intact, providing an explanation for persistent viral infection. The decline in barrier function coincided with the disruption of tight junctions, suggesting that chronic loss of barrier function caused by even subclinical CVB3 infection may increase susceptibility to neurological disease.

It is also known that a critical role in the regulation of viral infections and antiviral responses may be played by miRNAs. Coxsackievirus A10 (CV-A10) is a major cause of hand, foot, and mouth disease, but it is also responsible for cases of aseptic meningitis. The central players in the pathogenesis of CV-A10-induced CNS complications are still unknown. Using high-throughput sequencing Hu et al. identified differentially expressed miRNA in human umbilical vein endothelial cells (HUVECs) following infection with CV-A10. In their study several regulations related to the neurosystem were identified that may contribute to the neuropathogenesis of the CV-A10 infection. Significantly, miR-143-3p, a miRNA with a proven role in CNS diseases, was found among the dysregulated miRNAs. In conclusion, miR-143-3p may negatively impact BBB integrity during infection with CV-A10.

Several bacterial species are neuroinvasive and can cross the blood-brain barrier. While *Neisseria meningitidis* (*N. meningitidis*) causes acute meningitis, *Borrelia bavariensis* (*B. bavariensis*) leads to chronic neurological manifestations. To identify differences in the host cell response to the two bacterial species, Kulkarni et al. infected human BBB spheroids with *N. meningitidis* and *B. bavariensis* and investigated the response of the BBB cells. Both pathogens compromised the BBB integrity of the spheroids. The study characterized differentially expressed genes by transcriptome analysis and revealed that the regulation of certain molecular events by the two pathogens, such as interferon signaling, differed. In total, 48% of the differentially expressed genes were inversely expressed following challenge with *N. meningitidis* or *B. bavariensis*, offering opportunities for further investigations targeting the differences between the two bacteria.

Parkinson’s disease (PD) represents the second most prevalent neurodegenerative disorder with motor and cognitive impairments associated with BBB leakage. Over the last decade, the pathogenesis of PD has been associated with neuroinflammation and Glucose-6-Phosphatase-Dehydrogenase (G6PD) dysfunction compared to healthy individuals. An increased frequency of periodontitis (PDD), an inflammatory disease affecting the tooth-supporting structures caused by the host’s response to periodontal pathogens, has been reported in long-term PD-affected patients, but the missing link is not yet understood. Laugisch et al. evaluated the association between PD and PDD. In their retrospective study, a bidirectional relationship between PD severity, motor and cognitive impairment, and periodontal pathogens in saliva was confirmed. Lower G6PD activity was detected in the saliva of PD patients, indicating that G6PD dysfunction may impair the antioxidant response to inflammation-associated oxidative stress in these patients, as a modulating association between PD and PDD.

Among the pathogens that infect the CNS, fungal infections cause very high morbidity and mortality. The most prevalent pathogen responsible for fungal brain infections is *Cryptococcus neoformans* (*C. neoformans*). Lanser et al. summarized the evidence for the involvement of the BBB during brain invasion by *C. neoformans* due to direct interplay between the pathogen and the brain endothelium. They especially considered the potential role of macropinocytosis during internalization and transcytosis. They proposed a binary pathway mediated by the Ephrin receptor tyrosine kinase, EphA2, involving a macropinocytic transcellular pathway (mediated by EphA2 signaling) and a paracellular pathway (involving EphA2-mediated tight junction remodeling).

In summary the articles published in the Research Topic *“Host-pathogen interactions at the blood-brain barriers“* provide insights into the multiple processes involved in the entry of pathogens as diverse as viruses, bacteria and fungi at the blood-brain barrier into the brain. A deeper understanding of these host-pathogen interactions will help to understand the pathogenesis and pathophysiology of CNS diseases caused by pathogens and develop treatment options. In parallel, more and more research data report on the relationship between pathogen-associated inflammation and the pathogenesis of several neurodegenerative diseases, such as Parkinson’s Disease, Multiple Sclerosis, or others, arguing for further investigation. The manuscripts published here mainly cover processes at the BBB, but certainly an increased emphasis on research also on the BCSFB at the CP and the arachnoid mater would further contribute to the knowledge of this important topic.

## Author contributions

CS: Writing – original draft. CC: Writing – review & editing. SM: Writing – review & editing. XW: Writing – review & editing.

